# Hymecromone Promotes Longevity and Insulin Sensitivity in Mice

**DOI:** 10.3390/cells13201727

**Published:** 2024-10-18

**Authors:** Nadine Nagy, Kathryn S. Czepiel, Gernot Kaber, Darko Stefanovski, Aviv Hargil, Nina Pennetzdorfer, Robert Targ, Saranya C. Reghupaty, Thomas N. Wight, Robert B. Vernon, Rebecca L. Hull-Meichle, Payton Marshall, Carlos O. Medina, Hunter Martinez, Anissa Kalinowski, Rudolph D. Paladini, Stavros Garantziotis, Joshua W. Knowles, Paul L. Bollyky

**Affiliations:** 1 Division of Infectious Diseases and Geographic Medicine, Department of Medicine, Stanford University, Stanford, CA 94305, USA; nnagy@stanford.edu (N.N.); kczepiel@stanford.edu (K.S.C.); ahargil@gmail.com (A.H.);; 2 Department of Clinical Studies, School of Veterinary Medicine, University of Pennsylvania, Kennett Square, PA 19348, USA; sdarko@vet.upenn.edu; 3 Department of Pathology, School of Medicine, Stanford University, Stanford, CA 94305, USA; 4 Benaroya Research Institute, 1201 9th Ave, Seattle, WA 98101, USArvernon@benaroyaresearch.org (R.B.V.); 5 Division of Metabolism, Endocrinology and Nutrition, Department of Medicine, VA Puget Sound Health Care System and University of Washington, Seattle, WA 98108, USA; rhullmeichle@ualberta.ca; 6 Halo Biosciences, 125 University St., Palo Alto, CA 94301, USArudy@halobiosciences.com (R.D.P.); 7 Immunity, Inflammation and Disease Laboratory, Division of Intramural Research, National Institute of Environmental Health Sciences, Research Triangle Park, Durham, NC 27709, USA; garantziotis@niehs.nih.gov; 8 Cardiovascular Medicine and Cardiovascular Institute, School of Medicine, Stanford University, Stanford, CA 94305, USA

**Keywords:** hymecromone, longevity, insulin resistance, glycemic control, hyaluronan

## Abstract

Given that the extracellular matrix polymer hyaluronan (HA) has been implicated in longevity, we asked whether 4-methylumbelliferone (4-MU), an inhibitor of HA synthesis, impacts lifespan in mice. We designed a prospective study of long-term administration of 4-MU with conventional C57BL/6J mice. We find that 4-MU extends median survival from 122 weeks (control) to 154 weeks (4-MU), an increase of 32 weeks (*p* < 0.0001 by Log-rank Mantel Cox test). The maximum lifespan of 4-MU treated mice increased from 159 to 194 weeks. In tandem with these effects, 4-MU enhances insulin sensitivity, a metabolic parameter known to regulate lifespan, as measured by insulin tolerance testing (ITT) as well as frequent sampling intra venous glucose tolerance tests (FSIVGTTs). We further observed that 4-MU treated mice weigh less while consuming the same amount of food, indicating that 4-MU treatment alters energy expenditure. However, we do not observe changes in tissue HA content in this model. We conclude that 4-MU promotes insulin sensitivity and longevity but that the underlying mechanism, and the contribution of HA is unclear. 4-MU, already approved in various countries for hepatobiliary conditions, is currently under investigation and clinical development as a therapy for several chronic inflammatory conditions. These data suggest that the beneficial effects of 4-MU on tissue metabolism may include effects on longevity.

## 1. Introduction

Aging greatly impacts the quality and duration of human life. It is expected that by 2050, the number of people aged 60 years worldwide will reach 2 billion [1]. Aging is associated with many life-shortening pathologies, including insulin resistance. There is growing demand for new strategies that address the burden of the aging process.

Insulin resistance increases with aging [2]. In adults with normal glucose tolerance, there is a parallel increase in insulin resistance with aging, often associated with central obesity [3,4]. The most common age-related disorders associated with insulin resistance include type 2 diabetes (T2D), cardiovascular disease, and neurodegenerative diseases [5,6,7,8,9]. Although insulin is an essential hormone for growth, multiple findings suggest that elevated insulin levels promote age-associated diseases and shorten human lifespan [2].

The microenvironment surrounding cells in various organs, also referred to as the extracellular matrix (ECM), plays a decisive role in both insulin resistance and other characteristics of aging tissues [10]. Studies of aging tissues have shown that there are distinct and specific changes that occur in the ECM [11,12,13,14].

One molecule that is abundant in inflamed ECM and has been implicated in both aging and insulin resistance is hyaluronan (HA), a long, non-sulfated glycosaminoglycan. There are three HA synthases (HAS1-3) that catalyze HA synthesis from two activated sugar precursors, UDP-glucuronic acid (UDP-GlcUA) and UDP-N-acetyl-glucosamine (UDP-GlcNAc) [15]. In healthy tissues, HA is present within basement membranes and throughout the ECM. In inflamed tissues, pro-inflammatory cytokines [16,17] and hyperglycemia [18,19] drive HA production and catabolism, leading to the accumulation of HA fragments [20,21]. This process has been shown to drive cellular activation, migration, and proliferation [22].

While HA is transiently increased in many injured tissues, persistent HA deposits characterize settings of chronic inflammation [21] and immune dysregulation [23]. We and others have reported that ECM modulation of HA occurs in T2D models, including substantive increases in HA in skeletal muscle and adipose tissue [24]. Consistent with this, both HA and its receptor CD44 are implicated in insulin resistance [23]. In humans, serum CD44 levels are positively correlated with insulin resistance and glycemic control [21]. HA is upregulated in adipose tissue of obese mice and obese patients with T2D [25,26]; it also modulates adipogenesis and the inflammatory status of adipose tissue [19,23,27].

HA is also implicated in lifespan. In the human body, aging correlates with a decrease in HA content [28]. It has been shown in human fibroblasts that the downregulation of HAS2 seems to be linked with cellular senescence and aging. In studies on the longest-living rodent called the naked mole-rat (which can live for 40 years), the size and amount of HA are implicated in aging [29]. The very high molecular weight HA (vHMW-HA) (>6.1 MDa) protects cells from stress-induced cell-cycle arrest and cell death in a polymer length-dependent manner [29]. Implicating vHMW-HA in anti-aging mechanisms and suggesting the potential applications of vHMW-HA for enhancing cellular stress resistance [30]. On the contrary, aging is associated with the accumulation of HA degradation products. In the human body, aging correlates with a decrease of HA content [28]. In human fibroblasts, downregulation of HAS2 seems to be linked with cellular senescence and aging [13]. Notably, turnover of HA in tissues is significantly delayed for HMW-HA compared to HA fragments [31]. This suggests that inhibition of de novo HA synthesis may preferentially impact the amount of HA fragments, which are cleared faster, and shift the balance towards homeostatic HMW-HA. However, it is unclear how inhibition of HA synthesis might impact aging pathways in mice or humans.

Here, we examined how 4-methylumbellierone (4-MU), a well-established inhibitor of HA synthesis [22], impacts longevity and insulin sensitivity in C57Bl6 mice. 4-MU is thought to inhibit HA production in at least two ways. First, 4-MU is thought to function as a competitive substrate for UDP-glucuronyltransferase (UGT), an enzyme involved in HA synthesis [32], thereby depleting the cytosolic UDP-GlcUA pool [30]. In turn, the HA precursors GlcUA and GlcNAc accumulate and might be directed into other catabolic pathways. Second, 4-MU reduces expression of HAS mRNA [32] as well as mRNA for UDP glucose pyrophosphorylase and dehydrogenase [15]. The role of 4-MU in the inhibition of HA synthesis has been well established in multiple cell types and tissues [30,32,33]. We previously reported that short-term 4-MU treatment was associated with a modest increase in HA size [34], presumably due to enhanced clearance of HA fragments.

We find that the inhibition of HA synthesis with 4-MU extends the lifespan of mice, reduces blood glucose, and enhances insulin sensitivity, a metabolic parameter known to regulate lifespan.

## 2. Methods

**Animals:** Male C57BL/6J mice (at 4–5 weeks) were purchased from Jackson Laboratories. All mice were maintained in specific pathogen-free AAALAC-accredited animal facilities at Stanford University and handled in accordance with institutional guidelines.

**4-MU treatment:** A single control and 4-MU treatment group was established with 25 animals in each group. For the treatment arm, 4-MU (Alfa Aesar, Ward Hill, MA, USA) was pressed into the mouse chow by TestDiet^®^ as previously described [35]. In particular, mice were fed chow containing 5% 4-MU, a dose calculated to deliver 250 mg/mouse/day. This yielded a plasma drug concentration of 640.3 ± 17.2 nmol/L in mice, as measured by HPLC-MS [36]. The control diet had the same composition as the 4-MU diet without 4-MU. The 4-MU diet is formulated to have 3.47 kcal/g, and 3.46 kcal/g for the control diet. Mice were initiated on the 4-MU chow at 4–5 weeks of age, unless otherwise noted, and were maintained on this diet until they died of old age or were euthanized.

**Weight and diabetes monitoring:** Beginning at 4 weeks of age, mice were weighed weekly as well as bled via the tail tip puncture for the determination of their blood glucose concentration using a glucose meter and blood glucose monitoring strips (ReliOn PRIME (Wal-Mart Stores, Inc., Bentonville, AR, USA)). When two consecutive blood glucose readings of 300 mg/dL were recorded, animals were considered diabetic.

**Blood glucose measurements, IPGTT, ITT:** For these studies, we used protocols well-established in our lab [35,37]. In brief, mice were bled via the tail tip puncture for the determination of their blood glucose concentration using a glucose meter and blood glucose monitoring strips (Relion, Bayer). For IPGTT, mice were fasted 16 h overnight and given i.p. 2 g of glucose/kg body weight in PBS. For ITT, mice were fasted for 4 h and given i.p. 0.75 U/kg insulin. Blood glucose values were measured before and after glucose and insulin administration at 0, 15, 30, 60, and 120 min. The glucose meter used in this study has an upper threshold of 600 mg/dl. Therefore, values ≥600 mg/dL were diluted twofold and repeated.

**FSIVGTT:** A previously published protocol for performing insulin clamps on conscious, unrestrained mice was followed for the catheter insertion [38]. In brief, one catheter is inserted into the jugular vein for infusions. A second catheter is inserted into the carotid artery, which allows for blood sampling without the need to restrain or handle the mouse. This technique provides a significant advantage to the most common method for obtaining blood samples during clamps experiments, which is to sample from the severed tip of the tail. Unlike this latter method, sampling from an arterial catheter is not stressful to the mouse [39]. The FSIVGTT was performed in 5-h fasted B6 mice treated with 4-MU or control chow on day 7 following i.v. cannulation arterial and venous catheterization. Blood sampling was performed via the arterial catheter in unrestrained conscious animals. A baseline fasted blood sample was taken at –10 and 0 min. Based on a previous protocol, a 1 g/kg bolus of 50% dextrose was injected i.v. over a period of 15 s at t = 0 min. Blood (20 μL) was sampled for measurement of glucose and subsequent assay of plasma insulin at time points 1, 2, 4, 8, 12, 16, 20, 30, and 60 min. Additional samples were obtained for glucose measurement alone at 3, 5, 6, 10, 14, 18, 25, 40, and 50 min. Throughout the entire procedure, beginning at −10 min, mice received i.v. infusion of saline-washed erythrocytes (5 μL/min) to prevent the significant fall in hematocrit that would otherwise occur. Blood glucose levels were measured on a handheld glucometer (Accu-Check (Roche Diabetes Care, Indianapolis, IN, USA)) [40].

**Serum Insulin ELISA:** Blood was collected from mice via tail vein incision and using heparinized capillary tubes (BD Biosciences, Milpitas, CA, USA). The blood was centrifuged at 3000× *g* for 15 min at RT. The supernatant was collected and serum insulin concentrations were determined in triplicate using a rat/mouse insulin Enzyme-Linked Sorbent Assay (ELISA) kit from Millipore (EZRMI-13K, MilliporeSigma, Burlington, MA, USA) according to the instructions of the manufacturer. Rat insulin of 0–20 ng/mL was used as standard.

**Plasma analysis**: Blood was collected by heart puncture and anti-coagulated with 100 mM EDTA in isotonic sodium chloride solution. Plasma was prepared via centrifugation at 850× *g* for 15 min at 4 °C and stored at −20 °C for later analysis. Total cholesterol and triglycerides were subsequently quantified using the enzymatic in vitro tests Fluitest^®^ TG and Fluitest^®^ CHOL (Analyticon Biotechnologies, Lichtenfels, Germany).

**HA quantification:** HA quantification was performed as previously described. In brief, samples were first lyophilized and weighed, then were digested with proteinase K (250 µg/mL) in 100 mM ammonium acetate pH 7.0 overnight at 60 °C. After digestion, the enzyme was inactivated by heating to 100 °C for 20 min. Total amount of HA was determined by a modified competitive ELISA in which the samples to be assayed were first mixed with biotinylated HA-binding protein (b-HABP) and then added to HA-coated microtiter plates, the final signal being inversely proportional to the concentration of HA added to the biotinylated proteoglycan.

**Metabolic cages:** For the measurement and analysis of metabolism in mice, the Comprehensive Lab Animal Monitoring System for home cages (Columbus Instruments, Columbus, OH, USA) was used. The cages provide a sealed environment suitable for measuring Oxygen consumption and Carbon Dioxide production. The Oxymax cages are configured with dual axis detection of animal motion using IR photocell technology. Interruption of an IR beam will accrue one “count”. Coverage in a single plane may be implemented with IR photocells located in the X or XY direction. The height of these beams is such that they intersect the animal midway vertically. Also, by using an ultra-sensitive pressure transducer, changes in pressure produced by a subject respiring within the enclosed space produces cyclic variations of the pressure signal over time to reveal respiration frequency (breaths per minute) that could be detected.

**Statistical analysis:** Data are expressed as ± SEM of n independent measurements, unless otherwise noted. The comparison between 2 groups was performed with unpaired *t* tests. A *p* value less than <0.05 was considered statistically significant. Data analysis was performed with the use of GraphPad Prism 9.5.1 software.

## 3. Results

**Figure 1:**
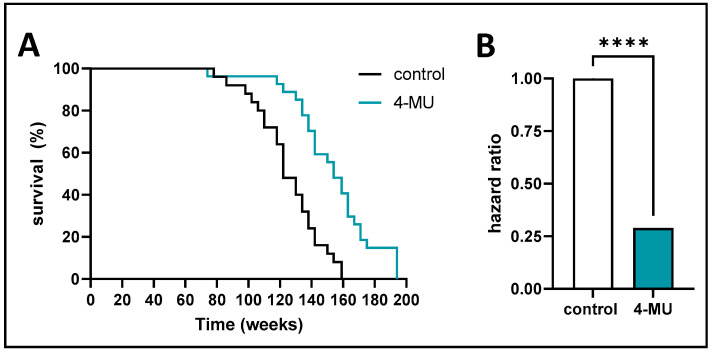
4-MU promotes longevity in mice. (**A**) Kaplan Meier survival curve of B6 mice treated with 4-MU and control chow. (**B**) hazard ratio calculation between 4-MU and control treatment. *n* = 20 mice per group. Data represent mean ± SEM; **** = *p* < 0.001 vs. control as calculated by unpaired *t* test.

**Inhibition of HA synthesis promotes longevity in mice.** We find that 4-MU extends the lifespan of C57BL/6J mice from 159 weeks (control) to 194 weeks (4-MU) max, with a median survival of 122 weeks (control) to 154 weeks (4-MU), resulting in a max increase of lifespan of 35 weeks, with a median increase of lifespan of 32 weeks (Figure 1). A hazard ratio of 0.29 (*p* < 0.0001) was calculated for the 4-MU treatment group compared to the control group, meaning there is a 71% relative risk reduction in death for mice treated with 4-MU (Figure 1). These data indicate that 4-MU treatment significantly prolongs the life of mice.

**Figure 2:**
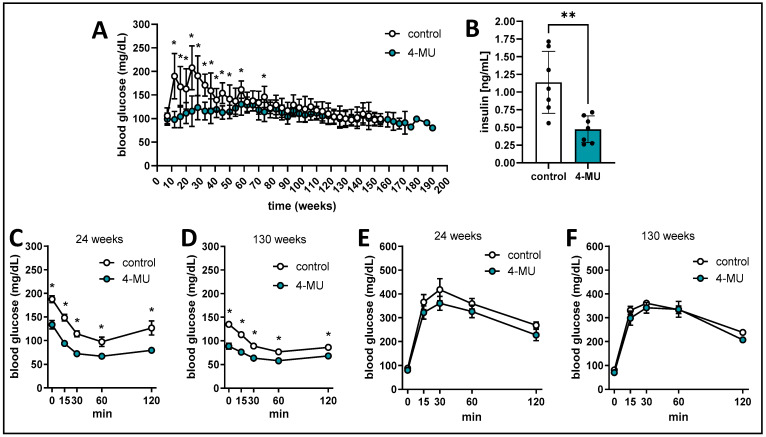
4-MU reduces blood glucose and promotes insulin sensitivity. (**A**) blood glucose measurements for the B6 mice in the survival experiment. (**B**) serum insulin measurement of 4-MU and control chow treated mice. (**C**,**D**) blood glucose during insulin tolerance test in 24 weeks old (**C**) and 130 weeks old (**D**) 4-MU and control chow treated mice. (**E**,**F**) blood glucose during glucose tolerance test in 24 weeks old (**E**) and 130 weeks old (**F**) 4-MU and control chow treated mice. (**A**) *n* = 20 mice per group. (**B**) *n* = 8 mice per group, (**C**–**F**), *n* = 4–8 mice per group. Data represent mean ± SEM; * = *p* < 0.01, ** = *p* < 0.003 vs. control as calculated by unpaired *t* test per timepoint.

**4-MU reduces blood glucose and promotes insulin sensitivity in mice.** We next examined the impact of 4-MU on the blood glucose of C57BL/6 mice. The mice started on 4-MU and control diet at 7 weeks of age and had a significant blood glucose reduction under 4-MU chow, which was most prominent in the first 60 weeks of treatment compared to control chow treated mice (Figure 2A). Despite the lower blood glucose, we also found a significant (*p* < 0.003) decrease of insulin under 4-MU treatment (control 1.137 ng/mL vs. 4-MU 0.4746 ng/mL ± 0.1804 ng/mL) (Figure 2B). Following up on this finding, we performed insulin tolerance tests (ITTs) and intraperitoneal glucose tolerance tests (IPGTTs) in 4-MU and control treated mice in cohorts of 24 and 130 weeks of age (Figure 2C–F). The ITTs of the 4-MU treated mice showed a higher insulin sensitivity compared to control mice independent of age (Figure 2C,D). It is notable that among untreated mice, 130-week-old animals had a lower blood glucose compared to 24-week-old animals. For the IPGTTs only a slight reduction in blood glucose could be seen under 4-MU treatment in the 24-week group (Figure 2E). No difference between the treatment groups in blood glucose could be detected in the 130-week-old mice (Figure 2F). There was no difference in liver enzymes (Supplemental Figure S1). These data indicate that 4-MU promotes insulin sensitivity in mice.

**Figure 3:**
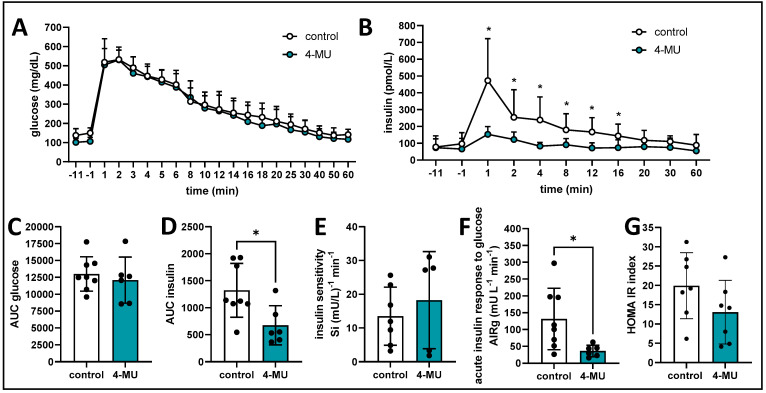
4-MU enhances insulin sensitivity in response to glucose challenge in mice. Analysis of Frequently Sampled Intravenous Glucose Tolerance Test (FSIVGTT) measurements in non-diabetic B6 mice treated with 4-MU and control chow. (**A**,**B**) glucose (**A**) and insulin (**B**) measurement for the duration of the FSIVGTT. (**C**,**D**) calculated glucose (**C**) and insulin (**D**) AUC for the duration of the FSIVGTT measurement. (**E**) insulin sensitivity expressed as SI, (**F**) acute insulin response to glucose expressed as AIRg. (**G**) approximate insulin resistance expressed as HOMA-IR. *n* = 6–8 animals per group. Data represent mean ± SEM; * = *p* < 0.05 vs. control as calculated by unpaired *t* test by timepoint.

**Inhibition of HA synthesis promotes insulin sensitivity in FSIVGTT.** After 4-MU’s promising data in insulin sensitivity, we further investigated the complex relationship between glucose and insulin. We performed a glucose tolerance test where glucose was infused via a carotid artery catheter and frequent blood glucose samples were taken during the time of the experiment via a catheter in the other carotid artery of the mouse. This investigational technique is known as a frequent sampling intra venous glucose tolerance test (FSIVGTT) [41]. We found that over the frequent sampling period of 60 min, the glucose values did not differ by treatment group (Figure 3A), whereas the insulin graph revealed a dramatic difference between treatment groups, showing a prominent insulin spike as reaction to the glucose infusion in control animals but no discernible change in 4-MU treated animals (Figure 3B). Calculation of the area under the curve (AUC) for glucose and insulin curves confirmed these observations (Figure 3C,D). Next, we analyzed the FSIVGTT data via the MINMOD Millennium software (MINMouseMOD version. 1.00), a computer program that calculates glucose effectiveness and insulin sensitivity from the FSIVGTT. We found that the insulin sensitivity is increased in 4-MU treated mice compared to mice on control chow (Figure 3E). We also found a significant decrease in the acute insulin response to glucose in the 4-MU treated mice when compared to the control group (Figure 3F). Finally, to investigate insulin resistance, we employed the Homeostatic Model Assessment for Insulin Resistance Index (HOMA IR), which demonstrated that insulin resistance was lower under 4-MU treatment (Figure 3G). Taken together, these findings point to a clear role for 4-MU in promoting insulin sensitivity and reducing insulin resistance.

**Figure 4:**
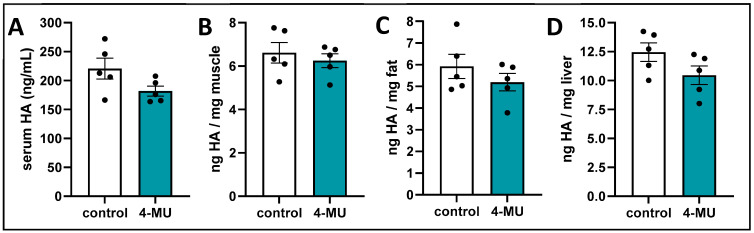
Long-term 4-MU does not reduce serum and tissue HA. (**A**) serum HA of 4-MU and control chow treated mice. (**B**–**D**) HA measurement in different mouse organs. HA measurement of 4-MU and control chow treated mice in muscle (**B**), fat (**C**), and liver (**D**). (**A**–**D**), *n* = 5 mice per group. Data represent mean ± SEM.

**4-MU slightly reduces serum and tissue HA concentration.** Because 4-MU inhibits HA synthases, and HA is prominent in insulin resistance, we examined the HA concentration of tissues that are known to play a role in insulin resistance. We examined tissues via HA ELISA and found only slight changes in HA between the treatment groups: serum (Figure 4A), muscle (Figure 4B), fat (Figure 4C), and liver (Figure 4D). This rather small effect might be explained by the fact that this study employed non-diseased C57BL/6J mice with overall low HA concentrations. These data let us wonder if the small decrease we saw in tissue HA content under 4-MU treatment is enough to promote insulin sensitivity, or if other mechanisms are involved.

**Figure 5:**
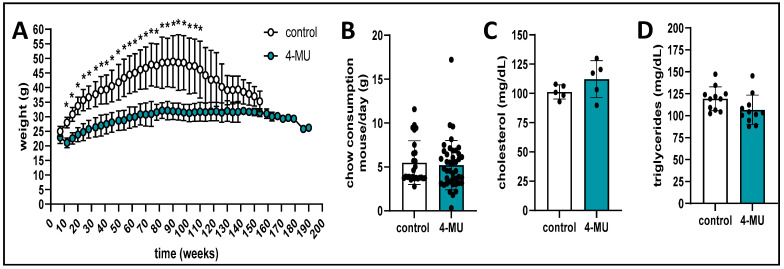
4-MU mice weigh less but eat the same. (**A**) weight measurements for the B6 mice in the survival experiment. (**B**) chow consumption per mouse per day over a 4 week period assessed in the survival experiment mice. (**C**,**D**) serum cholesterol (**C**) and triglycerides (**D**) of 15 week old B6 mice treated with and without 4-MU. (**A**,**B**) *n* = 20 mice per group. (**C**) *n* = 5 mice per group. (**D**) *n* = 11 mice per group. Data represent mean ± SEM; * = *p* < 0.05 vs. control as calculated by unpaired *t* test per timepoint.

**4-MU treatment reduces mouse weight while the food intake stays the same.** After we found that the tissue HA was only slightly reduced under 4-MU treatment, we looked at weight and chow consumption as well as blood cholesterol and triglycerides. All those parameters are known to be changed in T2D individuals exhibiting insulin resistance. We found that the mice under 4-MU treatment did not weigh as much as the control mice, with the difference being observed throughout the lifespan of the mice (Figure 5A). No difference in chow consumption between the treatment groups could be detected (Figure 5B). Analyzing the blood of the 4-MU and control treated mice revealed no difference in cholesterol (Figure 5C) and triglycerides (Figure 5D). Taken together, the data show that mice under 4-MU treatment weigh less despite comparable chow intake, suggesting potential effects of 4-MU on metabolism.

**Figure 6:**
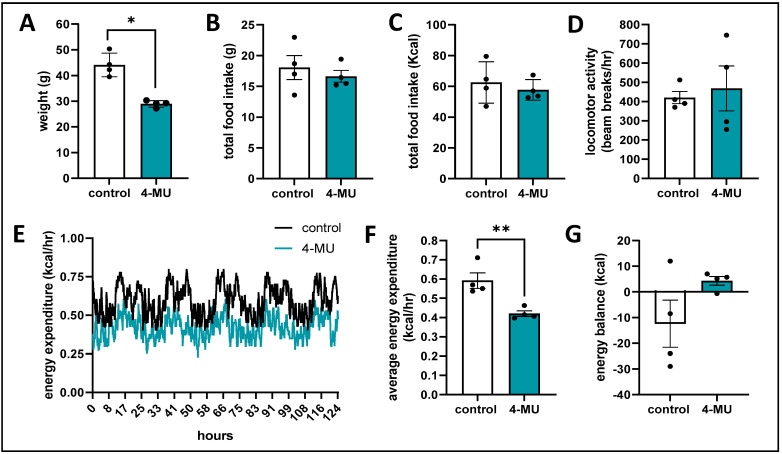
Metabolic cage mice weigh less, eat the same and have a lower energy expenditure. (**A**–**G**) analyses of metabolic cage experiment. (**A**) weight measurements for 9 months old C57BL/6J mice on 4-MU and control chow. (**B**) total food consumption in g (**B**) and kcal (**C**) of mice on 4-MU and control treatment. (**D**) locomotor activity of control and 4-MU treated mice. (**E**,**F**) energy expenditure as graph over time (**E**) and as average over time (**F**) normalized to body weight. (**G**) energy balance in kcal. Data represent mean ± SEM; * = *p* < 0.05 vs. control, ** = *p* < 0.007 vs. control as calculated by unpaired *t* test.

**4-MU treated mice exhibit a lower energy expenditure.** We investigated potential 4-MU effects on metabolism using metabolic cages. The mice were treated with 4-MU and control chow for 32 weeks before the start of the metabolic cage experiment. Metabolic testing confirmed that 4-MU treated mice had reduced weight (Figure 6A), while having similar food intake as control mice, measured both in gram (Figure 6B) and kcal (Figure 6C). We also investigated activity and movement of the mice and found no difference between groups (Figure 6D). We therefore measured energy expenditure, a measure for the total energy cost of maintaining constant conditions in the body plus the energy cost of physical activities. We found that 4-MU treated mice had a significantly lower energy expenditure compared to control mice (Figure 6E,F). Meaning that, 4-MU treated mice use less kcal/h, which in turn leads to a positive energy balance overall (Figure 6G). Taken together, those findings indicate that 4-MU-treated mice maintain a positive energy balance and eat the same as animals on conventional chow.

## 4. Discussion

We report that long-term treatment with 4-MU extends the lifespan of mice. These effects were potent and reproducible (we observed them in two different cohorts of mice). Moreover, the mice in this study appear to be healthier, with better serum lipid profiles and improved energetic profiles compared to mice on control chow. To our knowledge, this is the first report linking 4-MU treatment to longevity and healthfulness.

We also report that 4-MU increases insulin sensitivity. This work builds on our recent reports that 4-MU treatment reduces blood glucose in mouse models of both Type 1 Diabetes (an autoimmune disease) [33,34,42,43] and Type 2 Diabetes [33,44]. In those studies, we had attributed the beneficial effects of 4-MU on glycemic control to improvements in the number and phenotypic stability of insulin-producing beta cells in the pancreas [10,11]. The results presented here demonstrate that 4-MU also has substantial effects on insulin sensitivity.

The effects reported here are consistent with the hypothesis that 4-MU improves insulin sensitivity via effects on HA-dependent pathways. To this point, HA accumulates within multiple tissues in T2D leading to impaired insulin sensitivity [45,46]. In humans, both HA and its receptor CD44 are implicated in insulin resistance [45,47] and glycemic control [21]. Further, preclinical studies have shown that targeting CD44 with antibodies or nanoparticles protects against diet-induced weight gain and improves systemic insulin sensitivity [21,22,48,49]. HA is known to influence cellular metabolism and to contribute to cellular energetics by acting as a substrate for the hexosamine biosynthesis pathway. HA also has indirect effects on metabolism by driving inflammatory processes through TLR4 and NFkB responses. This raises the possibility that 4-MU impacts metabolism via effects on HA. However, despite the well-established reduction in HA levels seen with short term 4-MU [22], we only saw negligible differences in HA content in the serum, muscle, fat, and liver of mice that received long-term 4-MU, suggesting that HA production may reach a new equilibrium in the setting of long-term 4-MU. It is also possible that hypothetical off-target effects of 4-MU on other pathways [49] may be responsible. Further, detailed investigations of insulin signaling at the cellular level are needed to illuminate this biology. Unfortunately, those are beyond the scope of this present study. For now, we cannot conclude that these effects are HA dependent.

We propose that 4-MU effects on insulin sensitivity are a potential or partial explanation for how 4-MU extends lifespan given the established link between aging and insulin sensitivity [50,51,52]. Animal models of aging such as nematodes, fruit flies or mice have observed that decreased levels of insulin or insulin signaling promotes longevity [53,54,55]. However, these effects are complex and not always easy to compare between models. In *Caenorhabditis elegans*, the mutation of *daf-2* (an ortholog of the insulin receptor) is known to extend lifespan significantly, a finding that is somewhat surprising and not directly translatable to mice. Insulin receptor (IR) mutant mice display insulin resistance accompanied by hyperinsulinemia but do not show an extended lifespan compared to wild-type mice.

In humans, hyperinsulinemia and concomitant insulin resistance are associated with an elevated risk of age-related diseases [3,7,56,57]. Insulin resistance and impaired glucose tolerance increase with aging ([2,58]) even in individuals with normal body weight ([59]). Since impaired insulin resistance leads to aging [2], impaired insulin resistance and aging may form a feed-forward loop that accelerates the aging process. Our data suggest that 4-MU may inhibit this feedback mechanism and thus improve life and health span.

It is interesting to speculate how these results here might be related to reports that, very high molecular weight HA (vHMW-HA) promotes longevity in naked mole rats [29]. However, in our previous studies of the impact of 4-MU treatment on HA size and HAS synthase expression patterns, we did not see any evidence of vHMW-HA in mouse tissues [34,44,60,61,62]. One way to link these finding is the reduction of fragmentary LMW-HA we previously reported for animals that received 4-MU [34] for 1 month. Given the reports linking LMW-HA to pro-inflammatory effects, it may be that having lower levels of LMW-HA 4-MU achieves similar effects to vHMW-HA.

One confounding observation is the finding that mice given long-term 4-MU weigh less, as reported previously [60,63]. This unfortunately confounds the interpretation of 4-MU effects on longevity and insulin sensitivity, as calorie restriction and reduced body mass are also associated with these benefits. However, our data show that reduced caloric intake does not account for this difference. Consistent with an impact of 4-MU on energy expenditure, the metabolic cage experiments performed here demonstrated that 4-MU treated mice have a lower energy expenditure, as well as a higher energy balance compared to control mice. Consistent with this, in our previous studies, mice that were put on a diabetogenic diet and treated with 4-MU had improved glucose tolerance, insulin resistance, and enhanced mitochondrial function in brown adipose tissue (BAT) [64]. 4-MU alters cellular metabolism in ways that enhance mitochondrial function. Alternatively, it was recently reported that UDP-GlcUA could have negative effects on cancer cells. By lowering this sugar nucleotide, 4-MU theoretically could have potentially beneficial effects on the organism.

It is remarkable that the effects of 4-MU are reminiscent of metformin, a drug for treating T2D [65], which has been shown to enhance glucose control and insulin sensitivity [66,67] alongside with anti-aging effects at the cellular and organism levels [68], which are closely associated with improvements in aging hallmarks such as inflammation [69], autophagy [70], and cellular senescence [36,71,72]. We speculate that the similarity may hint at parallel underlying mechanisms. Future studies will investigate this relationship.

In conclusion, our data show that inhibition of HA synthesis with 4-MU extends the lifespan of mice, reduces blood glucose, and enhances insulin sensitivity. Excitingly, 4-MU is already an established therapeutic currently used in humans in Europe and Asia, to inhibit biliary spasm due to gallstones [49,50,51,52] with excellent safety record and tolerability [22,62,73]. 4-MU thus offers translational opportunities for the improvement of aging metabolism, health span, and lifespan. Further studies are needed to elucidate mechanisms that could be leveraged for lifespan extension in humans.

## Data Availability

The original contributions presented in the study are included in the article/supplementary material, further inquiries can be directed to the corresponding author.

## Supplementary Materials

The following supporting information can be downloaded at: https://www.mdpi.com/article/10.3390/cells13201727/s1, Figure S1: Long-Term 4-MU treatment does not impact liver function markers.

## Abbreviations

4-MU	4-methylumbelliferone
AUC	area under curve
BAT	brown adipose tissue
ECM	extracellular matrix
FSIVGTT	frequent sampling intravenous glucose tolerance test
GlcNAc	N-acetyl-glucosamine
GlcUA	glucuronic acid
HA	hyaluronan
HAS	HA synthases
HOMA-IR	homeostatic model assessment for insulin resistance
IPGTT	intra peritoneal glucose tolerance test
ITT	insulin tolerance test
RT	room temperature
T2D	type 2 diabetes
UDP	uridine diphosphate
UGT	UDP-glucuronyltransferase
vHMW	very high molecular weight
