# Supplementary material for: Hymecromone Promotes Longevity and Insulin Sensitivity in Mice
Source: Cells. 2024 Oct 18;13(20):1727. doi: 10.3390/cells13201727 (PMC11506560; doi:10.3390/cells13201727)
Supplement: Supplementary file 1 [file cells-13-01727-s001.zip › cells-3211933-supplementary.pdf]

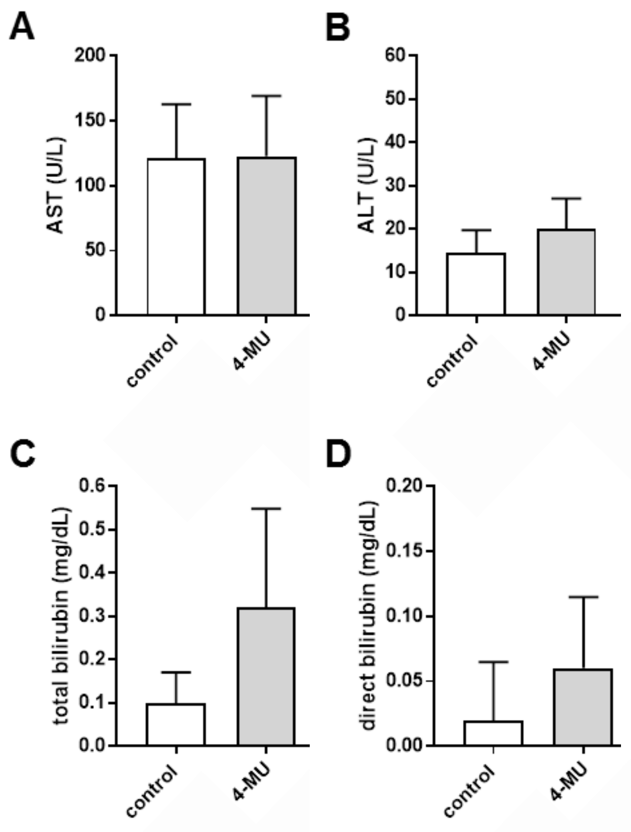

Supplemental Figure S1. long-Term 4-MU treatment does not impact liver function markers. Mice were treated with oral 4-MU in chow for 15 weeks. Each data point includes n = 5 mice.
